# Dislocation-free Ge Nano-crystals via Pattern Independent Selective Ge Heteroepitaxy on Si Nano-Tip Wafers

**DOI:** 10.1038/srep22709

**Published:** 2016-03-04

**Authors:** Gang Niu, Giovanni Capellini, Markus Andreas Schubert, Tore Niermann, Peter Zaumseil, Jens Katzer, Hans-Michael Krause, Oliver Skibitzki, Michael Lehmann, Ya-Hong Xie, Hans von Känel, Thomas Schroeder

**Affiliations:** 1 IHP, Im Technologiepark 25, 15236 Frankfurt (Oder), Germany; 2 Electronic Materials Research Laboratory, Key Laboratory of the Ministry of Education & International Center for Dielectric Research, Xi’an Jiaotong University, Xi’an 710049, China; 3 Dipartimento di Scienze, Università Roma Tre, Viale Marconi 446, 00146 Rome, Italy; 4 Technische Universität Berlin, Institut für Optik und Atomare Physik, Straße des 17. Juni 135, 10623 Berlin, Germany; 5 University of California at Los Angeles, Department of Materials Science and Engineering, Los Angeles, CA 90095-1595, United States; 6 ETH Zürich, Labor für Festkörperphysik, Otto-Stern-Weg, 18093 Zürich, Switzerland; 7 BTU Cottbus-Senftenberg, Konrad-Zuse-Straße 1, 03046 Cottbus, Germany

## Abstract

The integration of dislocation-free Ge nano-islands was realized via selective molecular beam epitaxy on Si nano-tip patterned substrates. The Si-tip wafers feature a rectangular array of nanometer sized Si tips with (001) facet exposed among a SiO_2_ matrix. These wafers were fabricated by complementary metal-oxide-semiconductor (CMOS) compatible nanotechnology. Calculations based on nucleation theory predict that the selective growth occurs close to thermodynamic equilibrium, where condensation of Ge adatoms on SiO_2_ is disfavored due to the extremely short re-evaporation time and diffusion length. The growth selectivity is ensured by the desorption-limited growth regime leading to the observed pattern independence, i.e. the absence of loading effect commonly encountered in chemical vapor deposition. The growth condition of high temperature and low deposition rate is responsible for the observed high crystalline quality of the Ge islands which is also associated with negligible Si-Ge intermixing owing to geometric hindrance by the Si nano-tip approach. Single island as well as area-averaged characterization methods demonstrate that Ge islands are dislocation-free and heteroepitaxial strain is fully relaxed. Such well-ordered high quality Ge islands present a step towards the achievement of materials suitable for optical applications.

The integration of high quality crystalline Ge on Si substrates has recently attracted intensive interests for its promising applications in diverse fields. In the continuous scaling of the complementary-metal-oxide-semiconductor (CMOS)-based digital devices, known as “More Moore (MM)” approach, Ge on Si is used as the channel material in p-MOS field-effect transistors (p-MOSFETs) due to its high hole mobility^1^. In novel low-power devices like tunneling FET (TFET), Ge can enable steep swing operation^2^. On the other hand, high quality Ge is also of paramount interest for the integration of various optoelectronic devices on Si (light emitters^3^,^4^,^5^, photo-detectors^6^ etc.), following the More than Moore (MtM) approach.

The major hurdles towards high crystal quality heterostructure growth are represented by the lattice mismatch (4.2%) and the coefficient of thermal expansion (CTE) mismatch (130%) existing between the Ge heteroepilayer and the Si substrate^7^. The resulting strain accumulation may be plastically relaxed through the formation of detrimental defects, such as misfit dislocations and cracks, thus hindering the realization of high quality heteroepitaxial crystals^8^. Furthermore, the thermodynamic-equilibrium epitaxy of Ge on Si (001) follows the Stranski-Krastanov (SK) growth mode, resulting in the nucleation of randomly distributed, size inhomogeneous^9^, and highly Si-Ge intermixed^10^ self-assembled Ge islands on top of a ~0.5 nm-thick Ge wetting layer.

In view of their possible use in Ge-based devices, numerous solutions have been proposed to achieve spatial and shape control of these self-assembled Ge islands, such as pit-patterning^11^,^12^,^13^, SiGe buffer layers^14^,^15^,^16^,^17^, epitaxial oxide buffer layers^18^,^19^, and the use of surfactants^20^,^21^. Nevertheless, these methods did not efficiently prevent the Si-Ge intermixing. For example, the relatively high temperature of ~700 °C required for selectivity in the “pit-patterned” selective growth results in alloyed SiGe islands with high Si content (>50%)^11^,^12^,^22^.

On the contrary, selective epitaxy by using SiO_2_-covered, patterned Si wafers allows one to obtain Ge nano-islands that are i) highly ordered in predefined seed areas, ii) size homogenous, and iii) suffer limited SiGe intermixing due to the small amount of Si at the surface. In prior studies, we have shown the successful selective chemical vapor deposition (CVD) growth of plastically relaxed Ge nanoheteroepitaxial islands on Si nano-pillar^23^,^24^,^25^ or nano-mesa^26^,^27^. Fully-coherent Ge islands have also been demonstrated on Si/SiO_2_ Si nanomesas, upon exploiting a few-nm thick SiGe buffer layer^28^. However, all these CVD-based approaches resulted in strongly pattern-dependent growth dynamics, inherently linked to the loading effect mechanisms of atomic species diffusing on the silica growth-mask.

Here, we report on molecular beam epitaxy (MBE) selective growth of dislocation-free Ge nano-islands on Si nano-tip wafers, fabricated by using a CMOS compatible technology. By means of transmission electron microscopy (TEM) and synchrotron radiation grazing-incident X-ray diffraction (SR-GIXRD), we experimentally demonstrate the achievement of fully coherent Ge islands, featuring very low Si interdiffusion, very high selectivity in predefined nano-seed areas, narrow shape and size distribution. Supported by theoretical modeling, we could also define the process conditions leading to a pattern-independent selective growth.

## Results and Discussion

### Si-tip wafers characterization

**Figure 1:**
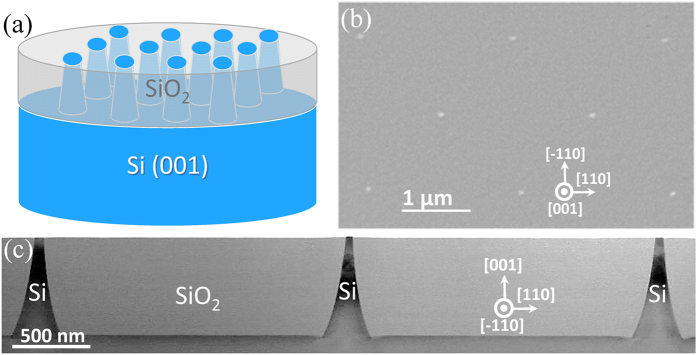
Si-tip wafer details. (**a**) An illustration of the wafer structure; (**b**) Plane-view SEM image for a Si-tip wafer showing a square pattern with its edge along Si [100] direction and tip-tip distance of 1.4 μm; coordinate system shows the orientations. (**c**) Cross-sectional TEM image of the same wafer displaying the tip-tip distance (along Si [110]) of 2 μm, Si-tip base diameter of ~300 nm and Si seeds diameter of ~40 nm; coordinate system shows the orientations.

Figure 1(a) illustrates the structure of Si-tip patterned wafers (more fabrication details can be found in Supplementary Information) while Fig. 1(b) shows a plane-view scanning electron microscopy (SEM) image obtained from a Si-tip substrate after chemical preparation, which reveals a perfectly periodic quadratic array of Si crystalline seeds (white dots) embedded in the SiO_2_ matrix (darker region) with a tip-to-tip distance of ~1.4 μm and the edge of the tip-square aligned along the Si [100] direction. In Fig. 1(c) we show the relative cross-sectional TEM image along the Si [110] direction. It displays that the diameters of the base and the opening of Si tips are ~300 nm and ~40 nm, respectively. The height of the Si tips is 800 nm. The SiO_2_ surface is atomically flat considering that the root mean square (RMS) roughness of the same wafer (after immersing in 0.5 wt.% HF for 10s) extracted from a 5 × 5 μm^2^ atomic force microscopy (AFM) image is only ~3 Å (see Figure S3 in Supplementary Information).

### Ge on Si-tip substrates: nucleation theory

In order to realize selective epitaxy, one must identify a “process window” in which the nucleation occurs only on the patterned Si nano-tip surface of the wafer. For instance, in the case of Ge selective CVD growth on pit-patterned Si substrates^29^, the selective growth is carried out at relatively high temperatures (>600 °C) and low deposition rates (0.1 ML/s or lower) in order to allow enough time for the Ge adatoms to diffuse to pit positions and nucleate there. In the Ge selective growth on Si-tip substrates, one has to find appropriate parameters to deposit Ge only on exposed Si crystalline seeds while avoiding random nucleation on the SiO_2_ surface. The desorption energy of Ge on SiO_2_ surface is ~0.44 eV^30^ while the sublimation energy of Ge from Si (001) has a much higher value of 4.25 eV^31^. Therefore, there exists a sizeable energy window allowing for the selective nucleation of Ge on Si over that on SiO_2_. To better investigate this point, in the following we discuss the selective nucleation phenomenon relying on an atomistic modeling of the process.

Let’s first discuss the nucleation of Ge on SiO_2_. According to the atomistic model of nucleation for heterogeneous growth, Ge atoms impinging on a SiO_2_ surface at a rate R either “stick” and diffuse on the surface or are re-desorbed. The diffusion of individual adatoms proceeds until they 1) bind to other atoms to form clusters, which are stable when the number of adatoms in the cluster reaches a critical value *i*; 2) are absorbed by existing stable clusters having a density n_x_; 3) diffuse into the substrate; or 4) are captured by defect sites of the substrate surface. Since the SiO_2_ surface layer of our samples can be considered as an “inert” and ideal substrate (see Supplementary Information), the processes 3) and 4) are not taken into account in the following. The key to the surface dynamics, i.e. the main features impacting the selectivity, is thus the value of n_x_, which is dependent on the rate R, the growth temperature T, the adatom desorption energy E_des_, the diffusion energy E_diff_, and the binding energy E_i_. For systems with different growth modes and different condensation regimes, n_x_ can be expressed in different forms. The growth mode of the Ge/SiO_2_ system is expected to generate three-dimensional clusters (3D) since


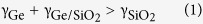


where γ_Ge_ (5 eV/nm^2^) and γ_SiO2_ (4 eV/nm^2^) are the Ge and SiO_2_ surface energy while γ_Ge/SiO2_ (10 eV/nm^2^) is the Ge/SiO_2_ interface energy, respectively^32^. The conditions required to achieve the adatom supersaturation needed for a stable growth, strongly depend on the property of the interface.

Three scenarios are usually observed. In the extreme incomplete regime (low E_des_ and low E_diff_), the adatom diffusion length^33^,^34^


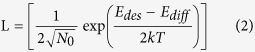


where N_0_ is the number of possible adsorption sites on the substrate and *k* is Boltzmann’s constant (1.3806 × 10^−23^ J/K), is much shorter than the distance


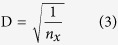


between stable clusters. As a consequence, the majority of adatoms desorb from the substrate surface. In the initial incomplete regime (high E_des_ and low E_diff_), we have L < D but the desorption time


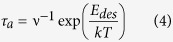


is relatively long (*v* is the characteristic surface vibration frequency in the range^33^ of ~10^11^ to 10^13^ s^−1^). Therefore, diffusing adsorbates, although partly staying on the surface, do not reach stable clusters either. On the contrary, in the complete condensation regime (extremely high E_des_ and/or extremely low E_diff_), the adsorbates can diffuse until they are captured by islands. Leonhardt *et al*.^35^ reported that Ge/SiO_2_ falls in the “extremely incomplete” regime even at deposition temperatures as low as 400 °C.

In this limit, the concentration of stable clusters n_x_ reads^8^,^33^:


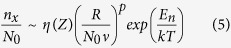


where *η*(*Z*) is coverage-dependent parameter varying from ~0.1 to 0.8 quasi-linearly when the island areal coverage Z increases from 10^−4^ to 0.4 (for extreme incomplete condensation and 3D island growth^33^. Here *η*(*Z*) = 0.1 was used but we note that with higher coverages, *η*(*Z*) and thus n_x_ is slightly greater); N_0_ is the number of possible adsorption sites on the substrate (~10^15^). The exponent *p* is related to the adatom number *i* in a critical cluster. The activation energy E_n_ (for the decay of nucleus) is related to E_des_, E_diff_ and E_i_. In case of the extreme incomplete condensation regime we have^33^


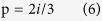


and


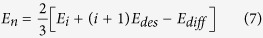


**Figure 2:**
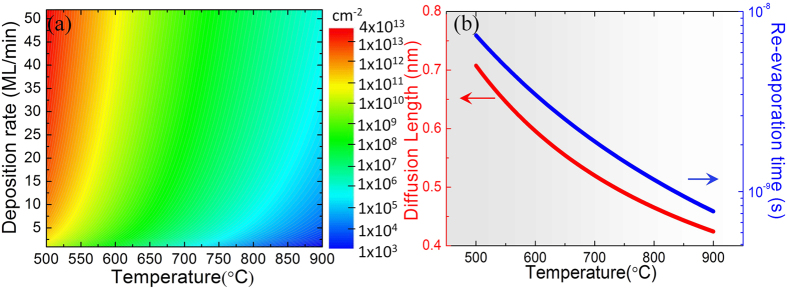
(**a**) A map of stable cluster density n_x_ of Ge adatoms on SiO_2_ surface as a function of deposition rate and temperature, calculated on the basis of an atomistic model of nucleation theory; colors from blue to red represent increasing density; (**b**) Dependences of diffusion length and re-evaporation time of Ge single adatoms over SiO_2_ on the substrate temperature.

Based on a spectroscopy study^30^, Li *et al*. found for the Ge/SiO_2_ system at T > 500 °C, i = 3, E_i=3_ = 3.7 eV, E_des_ = 0.44 eV, and E_diff_ = 0.24 eV (thus resulting in E_n_ = 3.48 eV). Using these values, we have plotted in Fig. 2(a) the concentration of stable Ge clusters on SiO_2_ surfaces, n_x_(R,T), as a function of the deposition rate R and temperature T. We observe that, the stable cluster density on SiO_2_ decreases for increasing T. On the contrary, as we pointed out above, the desorption energy E_des_ of Ge from Si is extremely high^31^ (about 10 times higher than E_des_ of Ge from SiO_2_ surface) and previous studies reported that high quality heteroepitaxial grown Ge could be realized on Si even at ~800 °C^29^,^36^. That is to say, Ge growth on Si falls in the complete condensation regime in the considered temperature range^37^. As a consequence, at sufficiently high temperatures where the nucleation of Ge on SiO_2_ surface can be almost completely avoided, Ge adatoms will only be adsorbed on top of the Si surface. In other words, at high enough temperatures, we have “super-saturated” conditions for the growth of Ge over Si whereas “under-saturated” conditions for that of Ge over SiO_2_. Thus the growth commences only on Si and the selective growth of Ge on Si-tip substrates is observed.

Figure 2(b) shows the dependence of the diffusion length and re-evaporation time of Ge single adatoms over the SiO_2_ surface as a function of substrate temperature. Since the Ge/SiO_2_ system has low E_des_ and E_diff_ (extreme incomplete regime), it features extremely short diffusion length and re-evaporation time. For example, at T = 750 °C, the Ge adatom diffusion length is ~0.5 nm only, while the corresponding re-evaporation time is ~1 × 10^−9^ s.

### Selective epitaxy of Ge on Si-tip wafers

We have thus shown that the high temperature and low deposition rate are predicted to favor the selective Ge growth on Si nano-tips. In this section, we will discuss the experimental growth results and compare them with the theoretical prediction.

**Figure 3:**
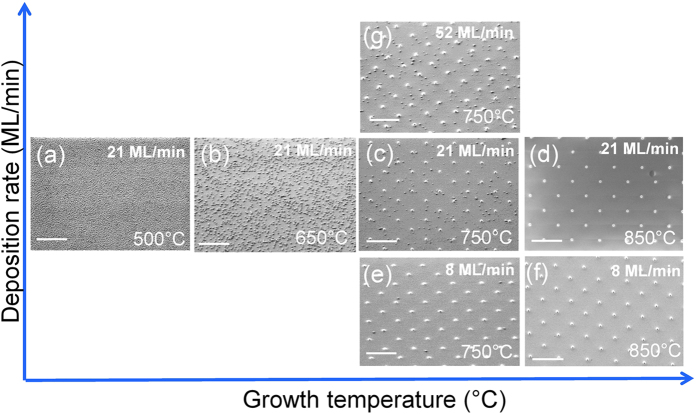
A morphology map (plane-view SEM images) of Ge/Si-tip samples grown under different conditions with the growth temperature as horizontal axis and deposition rate as vertical axis: (**a–d**) samples grown with 21 ML/min at 500 °C, 650 °C, 750 °C and 850 °C, respectively; (**e**) and (**f**) samples grown with 8 ML/min at 750 °C and 850 °C, respectively; and (**g**) sample grown with 52 ML/min at 750 °C. The scale bars in the images are all 2 μm and the condition details are marked on the SEM images.

We firstly discuss the morphology of a set of samples grown at different temperatures using R = 21 ML/min for the same time duration of 20 min. As displayed in Fig. 3(a), at T = 500 °C the growth results in a complete 2D Ge layer covering the entire substrate, confirmed also by *in-situ* X-ray photoemission spectroscopy (XPS) measurements (see Figure S4 in Supplementary Information). Upon increasing the process temperature, we observe the formation of Ge islands, at first randomly distributed on SiO_2_ (T = 650 °C, Fig. 3(b)) and then clustering around and on Si tip positions (T = 750 °C, Fig. 3(c)). At T = 850 °C (Fig. 3(d)), selective growth is achieved with almost no observed Ge island on the detected SiO_2_ surface area.

The samples grown at a lower deposition rate of 8 ML/min (Fig. 3(e,f)) show a similar temperature dependence as samples grown with 21 ML/min. In Fig. 3(f) for the sample grown at T = 850 °C with 8 ML/min, one finds no Ge island on the examined SiO_2_ surface area. If we now fix the growth temperature, say at T = 750 °C, and vary the deposition rate, it can be observed in Fig. 3(g,c,e) that lower deposition rate leads to lower Ge island density on the SiO_2_ surface. These results are qualitatively in good agreement with the theoretical prediction.

In order to quantitatively compare the experimental results with the nucleation theory presented in the Section 3.2, we report in Table 1 the calculated n_x_ and experimental results of Ge island density. It can be seen that the experimental values agree very well with the corresponding theoretical predictions. The slight discrepancy can be attributed to three main factors: 1) the coalescence of clusters. n_x_ in fact gives the maximum of the Ge island density on SiO_2_ under a certain growth condition and as a function of growth time, the Ge island density first reaches n_x_, then decreases due to island calescence; (2) the variation of the coverage (Z)-dependent factor η (Z)^33^: η (Z) = 0.1 was used for all calculations which is in fact more precise for lower coverages (e.g. Z ~ 10^−4^ for 850 °C samples), while for higher coverages (e.g. at T ≤ 750°C) it should have a larger value (but less than one order of magnitude) and thus the corresponding n_x_ should be slightly higher; and (3) particularly for 850 °C samples where very few Ge islands on the SiO_2_ surface can be detected, there is a relative large error bar of the experimentally measured Ge island density. As an example, for the sample grown at 850 °C and 8 ML/min shown in Fig. 3(f), while no Ge island is found on the examined SiO_2_ area, a single Ge island in the same area size would correspond to a density of ~7 × 10^5^ cm^−2^.

**Figure 4:**
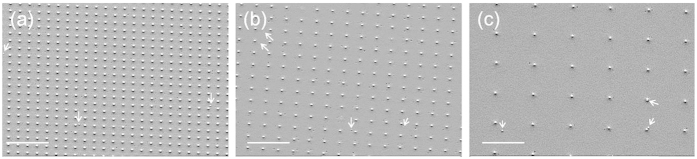
Plane-view SEM images of Ge islands grown on Si-tip wafers with different patterns. The growth conditions are the same as for the sample shown in Fig. 2(e), i.e. at 750 °C with 8 ML/min. The edges of the squares with tips at the corners are oriented along Si [110] and the tip-tip distances are (**a**) 0.5 μm, (**b**) 0.8 μm and (**c**) 2.0 μm, respectively. The scale bar is 2 μm for all images. The arrows mark a very few Ge islands observed on the SiO_2_ surface and the densities are almost the same for all patterns.

The selectivity of Ge islands grown on Si-tip wafers with different patterns was also examined to further elucidate the growth mechanism. Figure 4(a–c) shows samples grown under the same conditions as the sample shown in Fig. 2(e), i.e. T ~ 750 °C, deposition rate of 8 ML/min and duration of 60 min. Differently from the Si nano-tip pattern displayed in Fig. 2(e), here the Si tips are arranged in a square lattice oriented along the Si [110] direction and featuring tip-tip nearest neighbor distance of 0.5 μm, 0.8 μm, and 2 μm, respectively. We observe that, regardless of the particular tip distance, almost perfect selective growth is always achieved (see details of islands density summarized in Table 2). Independently from the pattern (tip lattice orientation, and Si seed density), the Ge island density on the SiO_2_ surface is thus independent from the Si nano-tip density (being a constant value of ~3 × 10^6^ cm^−2^), while the size and the volume of the Ge islands on different wafers are also the same. These results corroborate the growth mechanism described in nucleation theory 3.2 for our selective MBE growth, and exclude the occurrence of the “loading” effects typical for CVD growth.

### Dislocation-free Ge crystal: individual and global structures

**Figure 5:**
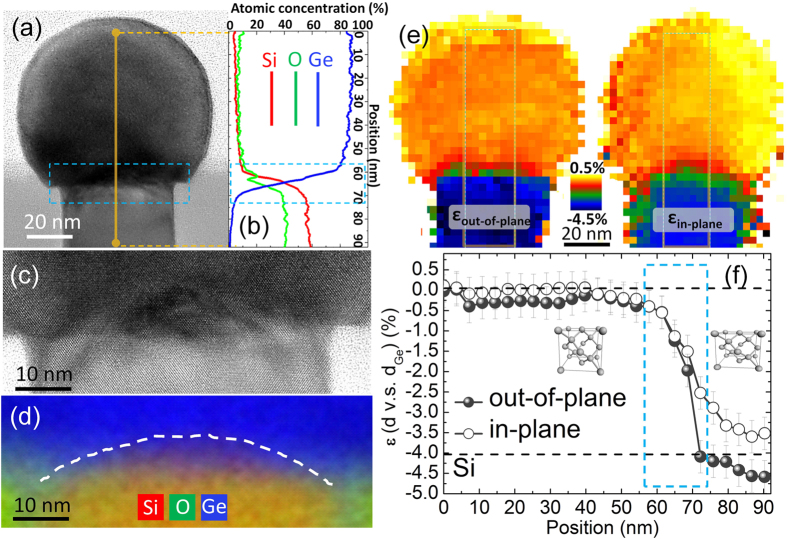
(**a**) A cross-sectional HRTEM image of Ge/Si-tip with (**b**) a corresponding EDX line profile along the orange straight line and red, green and blue curves represent Si, O and Ge respectively; An oxygen signal (~6%) was detected due to the surface oxidation of Ge in the TEM lamella, which therefore leads to the experimental measured Ge concentration (~88%). Because the oxygen species only locate on the Ge island surface, the real Ge concentration in the island bulk is as high as 93%. Blue dashed rectangles mark the interface region and its detail is shown in (**c**) by an enlarged HRTEM image and (**d**) a two dimensional (2D) EDX element map with Si (red), O (green) and Ge (blue) which shows the deformed top of the Si tip with an arc shape marked by the white dashed line. (**e**) 2D strain out-of-plane (left) and in-plane (right) maps of the Ge/Si tip measured with respect to the Ge lattice by TEM-NBD analysis. The relaxed Ge has ε = 0% and Si has ε = −4.0% (marked by black dashed lines); (**f**) strain line profile from top to bottom of the regions marked by rectangles in (**e**); the solid and empty circles correspond to out-of-plane and in-plane strain, respectively. The blue dotted rectangle marks again the similar interface region as in (**a**). Insets show relaxed Ge (left) and IP tensely strained Si (right).

Defects in heteroepitaxial Ge islands on Si mainly include interface defects, i.e. misfit dislocations (MD) and bulk defects, including point defects, threading dislocations (TD), stacking faults (SFs) and micro-twins (μ-twins). It is known that selective epitaxy on patterned wafers is an effective approach to reduce dislocations in lattice mismatch system^28^,^38^. Furthermore, because the lateral size of the substrate can be shrunk to nanometer size thanks to modern advanced lithography, the elastic relaxation of the growing material is significantly enhanced and strain partitioning between the epitaxial structures and the substrate could occur (so-called compliance). Therefore, the epitaxial structures can possibly undergo complete elastic relaxation with an infinite critical thickness for plastic relaxation^28^. According to a theoretical study^39^,^40^ of the Ge/Si system, Ge islands can be fully coherent on Si (001) when the lateral size of the Si seeds is below 50 nm, a limit greater than the tip opening diameter presented in this study (~40 nm). In Fig. 5 we show TEM measurements performed on an individual typical island belonging to the sample grown at T = 750 °C using R = 8 ML/min (see Fig. 4(a)). In the cross-sectional high resolution TEM (HRTEM) image of Fig. 5(a) we can observe the high crystalline quality of the Ge lattice showing no extended defects such as TDs or SFs/μ-twins. The nano island features nearly a hemispheric shape with tiny multi-facets^41^. An energy-dispersive X-ray spectroscopy (EDX) line profile analysis from top to bottom along the orange solid line was performed in order to examine the island composition (Fig. 5(b)). Surprisingly, the atomic concentration of Si in the core of the Ge island (s = [0–57] nm, in which “s” represents the position of the line profile) is ~6% only. Thus, the island consists of almost pure Ge even when grown at 750 °C, with limited intermixing occurring in the pedestal region only. This demonstrates an important advantage of Si-tip wafer approaches compared to other substrates, i.e. geometric intermixing hindrance. As a matter of fact, the pathway of Si inter-diffusion from the substrate is limited by the Si tips with nanometer size on surface: Ge islands on planar or pit-patterned Si substrates become Si rich (with Si concentration of >50%) when the temperature is above 650 °C^10^,^12^,^22^. We notice that an oxygen-related signal (~6%) was also detected in the Ge island bulk region, which is due to the surface oxidation of Ge in the TEM lamella, leading to the experimentally measured Ge concentration of ~88%. Because the oxygen species are only located on the Ge island surface, the real Ge concentration in the island bulk is as high as 93%. At the interface region (s = [57–72] nm, blue dotted rectangle), the Ge concentration decreases rapidly and reaches zero beyond ~80 nm.

Particular attention has been paid to the lattice quality in the interface region (marked by the blue dotted rectangle) in order to further examine the possible presence of MDs at the interface due to plastic relaxation of the nanometric islands. As we can see in the detailed HRTEM image shown in Fig. 5(c), no MD was observed, indicating that the strain induced by Ge-Si mismatch is entirely released through elastic relaxation, with the slightly intermixed, thin interface region acting as a graded buffer layer hindering plastic relaxation. Moreover, according to a statistical analysis carried out on more than 50 islands, the majority shows also no other defects, e.g. SFs or μ-twins. Only in a few islands, some SFs propagating from the shoulder of the Si tip were observed, which were probably formed owing to defects on the surrounding SiO_2_ “wall” surface^42^,^43^ (Supplementary Information, Figure S7). A color contrast can be noticed at the center of the Ge/Si interface (Fig. 5(c)), that can be attributed to a slight lattice distortion in this region. A detailed EDX element map was therefore performed and is shown in Fig. 5(d), which shows more interface details and helps to clarify the source of this lattice distortion (Si, O and Ge are represented by red, green and blue, respectively). We can observe that the top of the Si tip has a dome-like shape, (see white dashed line). This bowed geometry is probably due to the reaction of the Si tip atoms with the surrounding SiO_2_ at high temperatures. The thermally activated Si + SiO_2_→2SiO (g) reaction promotes the consumption of the tip apex through the formation of volatile SiO^44^. This phenomenon happens during the *in-situ* high temperature (>750 °C) pre-baking required to remove the native oxide prior to the Ge deposition (Supplementary Information).

During the epitaxial growth, Ge covers the Si-tip dome thus leading to the lattice distortion observed in Fig. 5(c). It is noted that a similar effect was observed for fully coherent Ge/SiGe/Si nanostructures on SOI wafers^28^. The dome shape of the Si tip covered by the Ge island, results in the projected atomic concentration at the interface (detected in Fig. 5(b)). In other words, the Si-Ge intermixing at the interface should be even weaker or the intermixing thickness should be even thinner. This highlights once more the geometric intermixing hindrance effect of the Si nano-tip wafer approach.

These results indicate that fully coherent, dislocation-free Ge crystals can be directly grown on Si thanks to three important mechanisms: 1) largely reduced Si substrate area with nanometer size (~40 nm in this case) yielding entirely elastic relaxation by 3D relaxation; 2) self-formed Si-Ge intermixing layer at the pedestal region of Ge islands and 3) possible compliance effect, i.e. strain partitioning between the Ge islands and the Si tip substrate.

In order to shed more light on the relevance of these three mechanisms we performed a detailed strain analysis of the island. In Fig. 5(e) we display the strain maps for both out-of-plane (OP, left panel) and in-plane (IP, right panel) strain. The OP and IP lattice parameters maps were extracted from nano-electron beam diffractions^45^ (NBD) (~5 nm diameter) taking into account (002) and (220) lattice points, respectively. Then strain maps were realized using a bulk Ge lattice constant of a_Ge_ = 5.658 Å as the reference. The relaxed Si_0.07_Ge_0.93_ lattice (corresponding to the island stoichiometry as detected by EDX) and Si have strain values of ε = −0.2% (orange) and ε = −4.0% (blue), respectively. It can be seen that in the core of the Ge island, both OP and IP maps show a similar value of strain, evidencing a completely relaxed cubic lattice. Some inhomogeneity in the strain maps of the Ge island, particularly in the IP map can be observed which is related to the technical difficulty in realizing perfect strain maps by using the TEM-NBD method. TEM samples usually are not ideal with exactly one orientation and the nanostructures are usually bent after the preparation of TEM samples, which lead to slight deviations of the diffraction spot positions thus the inhomogeneity in the strain maps. The green regions (with −3% < ε < −1%) observed at the interface in both OP and IP maps are transition regions. However, compared to the OP map, the green region in IP map is thicker and extends more to Si tip part. This can be better appreciated in Fig. 5(f), where we show a line profile of the region marked by rectangles in Fig. 5(e). Solid circles and empty circles represent OP and IP strain, respectively. The ε values for pure Ge and Si bulk are 0% and −4.0% (dashed lines in Fig. 5(f)), respectively. On the Ge surface and in the core of the Ge island, both OP and IP strains are very close to the expected value for entirely relaxed material (as shown in the inset at left part of Fig. 5(f)). They both decrease sharply at the interface (marked by the blue rectangle, similar region as in Fig. 5(a)) and reach their minima beyond the position of s ~ 70 nm. This behavior is strongly correlated to the similar rapid decrease of the Ge concentration in the same region. However, it can be observed that the IP strain decreases at a slightly slower rate than the OP counterpart, IP being larger than OP in the Si tip region, where the lattice is thus in-plane tensile strained. On one hand, this can be attributed to the presence of an intrinsic strain in the Si tips induced by the residual stress in tetraethylorthosilicate (TEOS)-CVD SiO_2_ surrounding the tip^46^,^47^. On the other hand, it possibly indicates a compliant strain partitioning in the vicinal region of the Ge/Si interface between the Ge nanocrystal and the Si nano-tip.

In order to better understand the strain relaxation of the Ge/Si-tip system, with particular focus on the possible compliance effect, SR-GIXRD in-plane measurements were performed in the vicinity of Ge and Si (220) Bragg peaks, providing information on the collective behavior of all Ge islands on Si tips in a spatially-averaged way.

**Figure 6:**
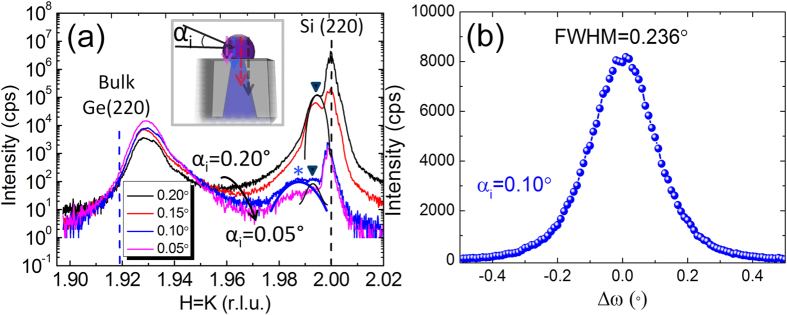
(**a**) SR-GIXRD H = K measurements with incident angles of X-ray beam α_i_ = 0.20° (black), 0.15° (red), 0.10°(blue) and 0.05° (magenta). Smaller α_i_ angles permit obtaining more signal from the interface and Ge islands, as shown by the inset scheme. Dashed lines at H = K = 2.000 (black) and H = K = 1.919 (blue) represent bulk Si (220) and Ge (220) peak positions, respectively. Black triangles mark the peak at H = K = 1.994 (fitted by a black peak) related to the intrinsic strain in Si tips induced by TEOS-CVD SiO_2_ and blue star (*) marks an additional peak at H = K = 1.987 (fitted by a blue peak) arising only for surface sensitive curves revealing probably a compliance effect (+0.6% IP tensile strain in pure Si). (**b**) ω scan of Ge (220) peak at H = K = 1.928 of α_i_ = 0.10° curve (blue) (FWHM = 0.236°).

Figure 6(a) shows the symmetric H = K measurements (derived from the in-plane 2θ_χ_-φ scan) obtained at different grazing incident angles (α_i_). For decreasing α_i_ the X-ray detection depth decreases and the technique probes sample regions closer to the surface, as shown in the inset illustration. When α_i_ = 0.20° (black curve), the X-ray penetrates deep into the Si-tip wafer; therefore a strong and sharp Si reflection from the Si substrate can be observed at H = K = 2.000 (black dashed line). Another strong but less intense peak appears at H = K = 1.994 (black triangle, fitted by a black peak), which can be attributed to the intrinsic IP tensile strain in the Si tips, as already observed in Fig. 5(f). The corresponding strain value is ε = +0.2%, which agrees very well with the intrinsic strain value measured in the Si-pillar patterned wafers which were fabricated similarly by TEOS-CVD SiO_2_^47^. The peak at H = K = 1.928 is related to Ge islands and perfectly matches the H = K value for a fully relaxed Si_0.07_Ge_0.93_ alloy. This indicates that no residual strain remains in the Ge island cores, in agreement with the strain analysis by TEM-NBD method (Fig. 5(e,f)). The continuous background existing between the Ge islands and Si-tip features the graded SiGe layer at the heterointerface.

Slightly decreasing the penetration of the X-ray beam (α_i_ = 0.15°) (red curve) leads to a significant decrease of the Si (220) peak intensity as well as that of the H = K = 1.994 Si peak, while the Ge reflection intensity increases. Another slight reduction (0.05°) of α_i_ to 0.10° induces an even more dramatic decrease (more than two orders of magnitudes) of Si (220) peak intensity compared to α_i_ = 0.15° case while the Ge (220) intensity again increases. More importantly, the Si (220) intensity is smaller than that of Ge (220), indicating that the α_i_ = 0.10° curve (blue) reveals mainly the information from the Ge/Si tip interface and Ge islands. It can be clearly seen in the α_i_ = 0.10° curve, that the main Si (220) peak slightly shifts to a lower H-K value (H = K = 1.999) and, in addition to the intrinsic strained Si tip peak (black arrow), a second broad Si shoulder appears at H = K = 1.987 (marked by a blue star and fitted by a blue peak, corresponding to a tensile strain of ε = +0.6%). For the Ge (220) peak, no shift is observed. When α_i_ = 0.05°, a similar behavior can been observed with increasing Ge signal but weaker Si related peaks.

Let us now consider the possible mechanism leading to the detected XRD curves. In selective epitaxy, besides 3D relaxation, the accumulated strain during the growth of Ge on Si (4.2% lattice mismatch) is released by either SiGe intermixing or strain partitioning, both of which result in a shift of Ge or Si peaks. It is difficult to disentangle the two effects. It is reported that multi-wavelength anomalous grazing incidence diffraction could be an effective method to address this issue^18^,^48^. Based on our single wavelength normal SR-GIXRD results, except the H = K = 1.994 Si peak due to intrinsic strain in Si tips (IP tensile, +0.2%), all additional peaks are explained in line with literature by SiGe intermixing. For instance, the wide shoulder at the right side of the main Ge (220) peak corresponds to a self-formed gradient SiGe layer. It follows that the H = K = 1.987 Si peak (indicated by *) could also be explained by a Si_0.83_Ge_0.17_ interfacial layer, if one does not take any strain partitioning into account. However, SiGe intermixing layers reported in literature exhibit a smooth, continuous grading increase from Si substrates to Ge epilayers or islands, the interpretation of this XRD peak by the presence of a “magic” composition Si_0.83_Ge_0.17_ is thus not favored. We consider that the H = K = 1.987 peak (*) is more likely due to the IP tensile strain (+0.6%) of the Si tips induced near the interface region by the overgrown Ge nanostructure: strain partitioning thus possibly occurs. This corroborates the TEM-NBD observations in Fig. 5(e). We note here that an increase of the intrinsic tensile strain of Si tips at the top region can also lead to the H = K = 1.987 peak. In this case, it is possible that the tensile strain of the Si tip induced from the surrounding TEOS SiO_2_ stressor^47^ increases to the top region due to the conical tip shape. An ω scan was performed at H = K = 1.928 (Ge island peak) of α_i_ = 0.10° curve (blue) to characterize the crystallinity of Ge islands, as shown in Fig. 6(b). It can be seen that the full width at half maximum (FWHM) of the Δω peak of Ge (220) of Ge islands is only 0.236° demonstrating good crystalline quality of such fully coherent Ge islands.

## Conclusion

In conclusion, dislocation-free Ge crystalline nano-islands have been obtained via selective MBE on Si nano-tip wafers. The nucleation behavior of Ge adatoms on the SiO_2_ surface was discussed in detail based on an atomistic model of nucleation theory. Theoretical calculations predict selective growth of Ge on Si nano-tip wafers occurs when the growth conditions are close to thermodynamic equilibrium, i.e. at high temperatures and low deposition rates, where arriving Ge adatoms only nucleate on the Si crystalline seed surface and all Ge adatoms on SiO_2_ re-evaporate. The theoretical prediction was subsequently well confirmed by experimental results and such a mechanism leads to pattern-independent selective growth without the well-known “loading effect” for CVD growth. Thanks to geometric intermixing hindrance effects of Si nano-tip wafers, high growth temperatures (>750 °C) induce very weak Si inter-diffusion in Ge islands (93% Ge). Compared to Ge islands on planar Si (001) substrates, a self-formed ultra-thin SiGe intermixing layer at the pedestal region of Ge islands and probable strain partitioning in the top of the Si tips were observed. Both of them facilitate the achievement of fully coherent Ge islands on Si nano-tip wafers. The high temperatures needed to achieve the selective growth are also beneficial to the crystal quality of Ge islands. These results provide more observations and deeper understanding of emerging phenomena in the important area of nano-scale heteroepitaxy^49^,^50^,^51^. Our method for dislocation-free Ge islands on Si wafers opens a novel pathway to realize Ge on Si suitable for optical applications. Furthermore, such an approach is highly relevant to Si-based applications based on the heterogeneous integration of other alternative compounds on Si (e.g. III–V, II–VI).

## Methods

### Si-tip wafers fabrication

Nano-tip patterned Si (001) wafers with a wafer size of 8 inches were fabricated using 0.25 μm complementary metal-oxide-semiconductor (CMOS) technology. A CVD SiO_2_ layer using tetraethylorthosilicate (TEOS) gas source was deposited to cover the ultra-sharp Si tips. The opening area of crystalline Si seeds can be precisely controlled by a chemical mechanical-polishing (CMP) process and wafers with Si tips of opening of ~40 nm in diameter were employed. More fabrication details of Si-tip wafers can be found in Supplementary Information (Figure S1) and ref. 52.

### Selective epitaxy of Ge islands

Ge growth was carried out by using molecular beam epitaxy (MBE) in a DCA chamber. Prior to the Ge growth, Si-tip wafers were chemically prepared by HF dipping and pre-baked to obtain a clean Si seeds surface without native SiO_2_ (see details in Supplementary Information). It is noted here that the preparation method was carefully optimized to “open” the crystalline Si seeds and to avoid over-etching of SiO_2_ or severe deformation of Si tips (see Supplementary Information). The Ge growth was performed at temperatures ranging from 500 °C to 850 °C and deposition rates from 8 monolayer per minute (ML/min) to 52 ML/min. The Ge deposition rate was calibrated by direct growth of Ge layers on Si planar substrates.

### X-ray photoemission spectroscopy

After Ge islands growth, the physical-chemical properties of the samples were *in-situ* examined without breaking the vacuum using a SPECS X-ray photoemission spectroscopy (XPS) system with Al K-α radiation at 1487 eV. The Fermi level was calibrated with respect to a gold sample.

### Scanning electron microscopy

The samples were *ex-situ* characterized by scanning electron microscopy (SEM) with a Zeiss Nvision equipment, in which the electron beam energy was selected as 1.5 keV in order to avoid the charge effect stemming from insulating SiO_2_ material.

### Transmission electron microscopy

The crystallinity, interface, strain status, and the Si-Ge intermixing of individual Ge island were studied by a FEI Osiris (operated at 200 kV) transmission electron microscopy (TEM) system equipped with components for scanning TEM (STEM) and energy-dispersive X-ray spectroscopy (EDX) measurements. High resolution TEM (HRTEM) measurements were also performed using the FEI TITAN 80–300 Berlin Holography specially operated at 300 kV.

### X-ray diffraction

SR-GIXRD measurements were performed at BM32 beamline of the European Synchrotron Radiation Facility (ESRF) with an X-ray wavelength of 1.127 Å.

### Theoretical calculation of nucleation

The condensation behavior of Ge adatoms on the SiO_2_ surface was theoretically evaluated by using an atomistic model of the nucleation phenomenon. The stable nuclei density on SiO_2_ was calculated as a function of growth temperature and deposition rate (i.e. impingement rate of arriving adatoms) while the diffusion length and re-evaporation time of Ge adatoms on SiO_2_ at different temperatures were predicted.

## Additional Information

**How to cite this article**: Niu, G. *et al*. Dislocation-free Ge Nano-crystals via Pattern Independent Selective Ge Heteroepitaxy on Si Nano-Tip Wafers. *Sci. Rep.* **6**, 22709; doi: 10.1038/srep22709 (2016).

## Supplementary Material

Supplementary Information

## Figures and Tables

**Table 1:** Theoretical Ge stable cluster density n_x_ (Th.) and experimental Ge island density (Exp.) on SiO_2_ grown under different growth conditions (unit: cm^−2^).

	**650 °C**		**750 °C**		**850 °C**	
	**Th.**	**Exp.**	**Th.**	**Exp.**	**Th.**	**Exp.**
8 ML/min	/	/	3.3 × 10^6^	3.5 × 10^6^	1.0 × 10^5^	< 7 × 10^5^
21ML/min	7.4 × 10^9^	5.0 × 10^9^	7.0 × 10^7^	1.6 × 10^8^	6.7 × 10^5^	< 7 × 10^5^
52ML/min	/	/	3.6 × 10^8^	3.4 × 10^8^	/	/

Considering the variation of η (Z) and the possible coalescence of the islands during growth, the experimental values are in good agreement with the calculated n_x_ values.

**Table 2:** Ge islands density on SiO_2_ of Si-tip substrate with different tip square edge orientations and tip-tip distances.

**Square edge orientation**	**Tip-tip distance (μm)**	**Si seed density (cm**^−**2**^)	**Ge islands density on SiO**_**2**_**(cm**^−**2**^)
Si [110]	0.5	4.0 × 10^8^	2.5 × 10^6^
Si [110]	0.8	1.6 × 10^8^	3.3 × 10^6^
Si [100]	1.4	5.1 × 10^7^	3.5 × 10^6^
Si [110]	2.0	2.5 × 10^7^	2.5 × 10^6^

Growth conditions are identical with 8 ML/min at ~750 °C.
