# Supplementary material for: Dislocation-free Ge Nano-crystals via Pattern Independent Selective Ge Heteroepitaxy on Si Nano-Tip Wafers
Source: Sci Rep. 2016 Mar 4;6:22709. doi: 10.1038/srep22709 (PMC4778127; doi:10.1038/srep22709)
Supplement: Supplementary Information [file srep22709-s1.doc]

**Supplementary information**

**Dislocation-free Ge Nano-crystals via Pattern Independent Selective Ge Heteroepitaxy on Si Nano-Tip Wafers**

**Gang Niu^1,2*^, Giovanni Capellini^1,3^, Markus Andreas Schubert^1^, Tore Niermann^4^, Peter Zaumseil^1^, Jens Katzer^1^, Hans-Michael Krause^1^, Oliver Skibitzki^1^, Michael Lehmann^4^, Ya-Hong Xie^5^, Hans von Känel^6^, Thomas Schroeder^1,7^**

^1^ IHP, Im Technologiepark 25, 15236 Frankfurt (Oder), Germany

^2^ Electronic Materials Research Laboratory, Key Laboratory of the Ministry of Education & International Center for Dielectric Research, Xi'an Jiaotong University, Xi'an 710049, China.

^3^ Dipartimento di Scienze, Università Roma Tre, Viale Marconi 446, 00146 Rome, Italy

^4^ Technische Universität Berlin, Institut für Optik und Atomare Physik, Straße des 17. Juni 135, 10623 Berlin, Germany

^5^ University of California at Los Angeles, Department of Materials Science and Engineering, Los Angeles, CA 90095-1595, USA

^6^ ETH Zürich, Labor für Festkörperphysik, Otto-Stern-Weg, 18093 Zürich, Switzerland

^7^ BTU Cottbus-Senftenberg, Konrad-Zuse-Straße 1, 03046 Cottbus, Germany

Corresponding author: ^*^Gang Niu, [coyhyde@gmail.com](mailto:coyhyde@gmail.com)

**1. Fabrication details of Si-tip wafers and preparation before Ge growth**

Figure S1 (a)-(c) show the process details of such patterned wafers: (a) ultra-sharp Si tips (diameter of ~300 nm at base) array with a square pattern were fabricated by reactive-ion etching (RIE) process on the masked wafers, in which the tip-tip distance can be simply controlled by varying masks; The height of Si tips ranges from 470 nm to 800 nm then (b) a chemical vapor deposition (CVD) with tetraethylorthosilicate (TEOS) gas source was performed to deposit a SiO_2_ layer completely covering the Si tips; followed by (c) a chemical-mechanical polishing (CMP) process to reduce the layer thickness thus opening circular Si “seeds” with a minimal diameter of 5-10 nm. In this study the seeds size is ~40 nm. Prior to the Ge growth, Si-tip wafers were chemically prepared to obtain a clean Si seeds surface without native SiO_2_: 1) 10s immersing in Piranha solution and 10s rinsing in de-ionized (DI) H_2_O; 2) 10s dipping in diluted HF (0.5 wt%) followed by a 10s rinsing in DI H_2_O and 3) a pre-baking at ~790°C for 5 min in ultra-high vacuum (UHV) MBE chamber with a base pressure of ~5×10^-10^ mbar.


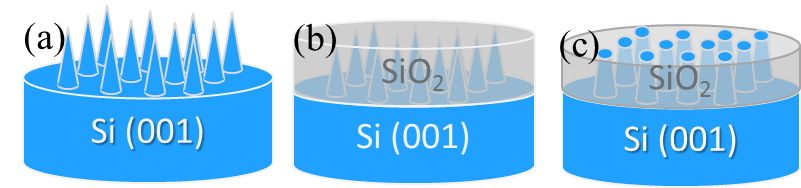


**Figure S1.** The fabrication process of Si-tip patterned wafers: (a) RIE etching of Si tips structure; (b) TEOS-CVD growth of covering SiO_2_ layer and (c) Si “seeds” opening by CMP.

**2. HF preparation for Si-tip wafers.**

Figure S2 shows cross-sectional TEM images in STEM-HAADF (High-Angle Annular Dark Field) mode of Si-tip wafers preparation with different HF (0.5%) treatments. The yellow dotted lines mark the profiles of SiO_2_ layers. Figure S2 (a) shows the result for a substrate with no HF immersion but annealed in UHV at 900°C for 5 minutes. Apparently, without HF immersion, the native SiO_2_ on Si tips cannot be removed even when the sample was annealed at a very high temperature of 900°C. Figure S2 (b) shows a substrate with 30s HF (0.5%) immersing and a subsequent 790°C annealing (5 minutes) in UHV MBE chamber. It can be observed that 30s HF immersing over-etches SiO_2_ with the Si tip sticking out of the SiO_2_ level. Therefore, the optimized HF treatment is 10s HF immersing, which removes native SiO_2_ but does not over-etch the large area SiO_2_ layer, as shown in Figure S3, which shows a 5×5 μm^2^ atomic force microscopy (AFM) image on a 10s HF immersed substrate. The sample surface is atomically flat with a RMS of only 3 Å. Furthermore, interestingly, ordered ring structure with very weak contrast (height different with surrounding surface less than 5 Å) can be observed, which is due to the structured Si tips embedded in SiO_2_ layer. It is possible that the Si tips strain fields slightly influence the HF etching rate of surrounding SiO_2_.


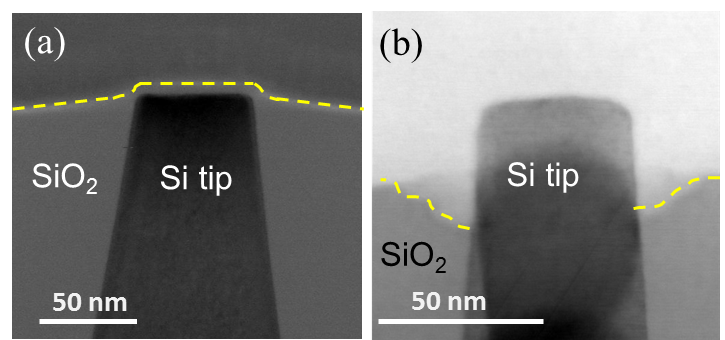


**Figure S2.** Cross-sectional STEM-HAADF images for substrates prepared by different methods: (a) annealing (5 min) at 900°C in UHV without HF immersing and (b) 0.5% HF immersing for 30s and a 5 min annealing at 750°C in a UHV-MBE chamber; Yellow dotted lines profile the SiO_2_ shapes.


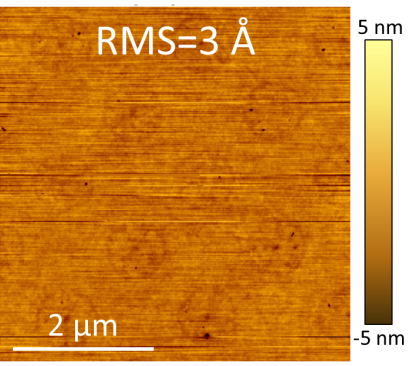


**Figure S3.** A 5×5 μm^2^ AFM image on a 10s HF (0.5%) immersed Si-tip wafer with RMS=3Å. The ring structure with weak signal is induced by the embedded patterned Si tips.

**3. In-situ XPS measurements for samples grown at different temperatures**

The samples grown with a growth rate of 21 ML/min at different temperatures were measured by means of in-situ XPS measurements with Al Kα excitation and results are shown in Figure S4 (a). The XPS intensity from Ge 2p (3/2) (binding energy BE=1218 eV) core level was analyzed owing to its limited escape depth (0.5 ML). For T=500°C sample (blue line), only the Ge 2p peak is visible while no Si spectral contibution is observed, indicating, in agreement with the observed morphology, that Ge completely covers the SiO_2_ surface. When the growth temperature was increased to T=650°C (orange), the intensity of Ge 2p peak strongly decreases compared to 500°C sample and a weak shoulder at ~1221 eV appears, revealing Ge-O bonds (originated from Ge islands on SiO_2_ surface, more details are shown in Figure S4 (b)). Moreover, a peak at BE=104.3 eV is observed, corresponding to the SiO_2_-related Si 2p peak, featuring a 4+ valence state. These results confirm that at T=650°C Ge islands do not cover the whole SiO_2_ surface. 750°C sample (rose) shows quite similar XPS result as 650°C: both Ge peak intensity and intensity ratio of
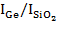
 decrease (from 1.17 to 1.02), indicating a lesser Ge islands footprint on SiO_2_. For the 850°C sample (red), the
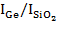
 ratio further decreases (to 1.00) and the 1221 eV shoulder of Ge 2p peak disappears, demonstrating that all detected Ge materials are on crystalline Si seeds and no Ge islands on SiO_2_ are observed.


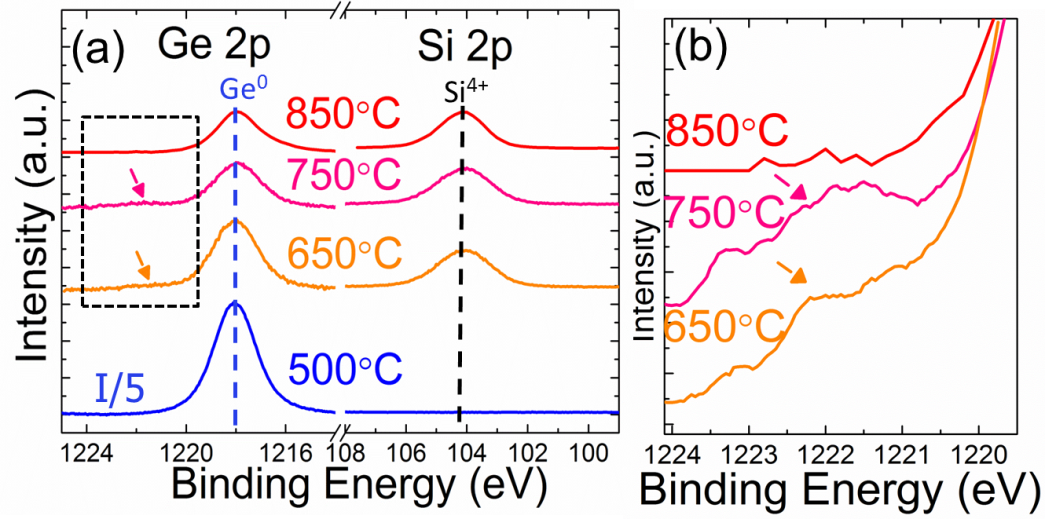


**Figure S4.** (a) In-situ XPS results of samples grown with 21 ML/min but at different temperatures (same samples as shown in Figure 3 (a)-(d)). Ge 2p (3/2) and Si 2p spectra are recorded and from bottom to top spectra correspond to 500°C (blue), 650°C (orange), 750°C (rose) and 850°C (red), respectively. The strong Ge 2p peak intensity of 500°C sample is divided by five. Dotted lines mark different valence positions of Ge and Si. The arrows mark weak shoulders of Ge peak (Ge-O bond) for 650°C and 750°C samples, induced by Ge islands on SiO_2_ surface. More details of this region (marked by the black square in (a)) are shown in (b).

**4. Impact of pre-annealing and post-annealing process on Ge islands quality**

It is found that both pre- and post- annealing processes strongly impact the quality of Ge islands. Figure S5 shows the STEM-HAADF images of Ge islands grown on a 750 °C pre-annealed Si-tip substrate. It can be observed in Figure S5 (a) that three Ge islands nucleate at different locations on the Si tip surface and an interfacial layer (white) can be seen between Ge and Si. Figure S5 (b) shows more details of the interface region marked by a yellow square in (a) and the arrow highlight clearly the existence of ~1 nm SiO_2_ layer at the interface. This can be attributed to the incomplete cleaning of native SiO_2_ layer on the Si tip surface, therefore the residual SiO_2_ (in nanometer size) hinders the nucleation of Ge islands. Furthermore, it is noticed that the separated Ge islands in (a) do not show any SFs or μ-twins. However, the coalescence of these islands leads to the formation of SFs and/or μ-twins, as shown in Figure S5 (c). The defects are marked by arrows.


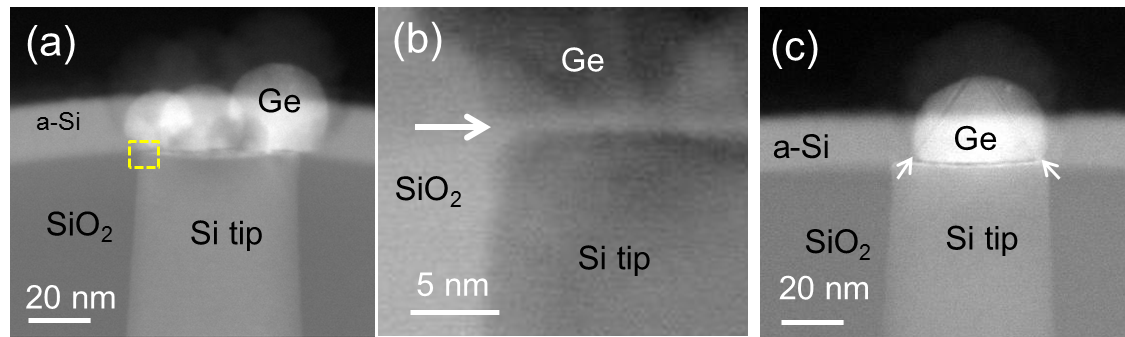


**Figure S5.** Cross-sectional STEM-HAADF images for Ge islands grown on a 750°C pre-annealed Si-tip wafer (covered subsequently by an amorphous Si protecting layer): (a) three SF-free Ge islands nucleated on the partly opened crystalline Si tip surface and the residual native SiO_2_ layer can be observed at the Ge/Si interface; (b) more interface details at the yellow squared region in (a) and the arrow highlights the ~1 nm SiO_2_ layer between Ge and Si tip; (c) coalesced Ge island demonstrating SFs/μ-twins marked by arrows.

Too high pre-annealing temperatures will also influence the Si tip and thus subsequently impact the Ge quality. Figure S6 (a) shows a cross-sectional STEM-HAADF image of a Si-tip substrate annealed at 900°C for 5 min in UHV. The Si tip top part reacts with surrounding SiO_2_ via the reaction Si+SiO_2_🡪2SiO (g) with volatile SiO products, thus resulting in a hole at the top region. Moreover, the top of the Si tip is also severely deformed to an irregular shape with sharp corners. Due to disordered Si tip surface and the SiO_2_ wall of the hole, the Ge island grown on this substrate (HRTEM image in Figure S6 (b)) shows many SFs and/or μ-twins. Details of the interface region (marked by a yellow square) are shown in Figure S6 (c), in which defects are marked by arrows. Therefore, a suitable pre-annealing temperature is required to prevent SFs/μ-twins at the initial growth stage. An optimized temperature of 790 °C was therefore employed for the samples repeated in the paper.


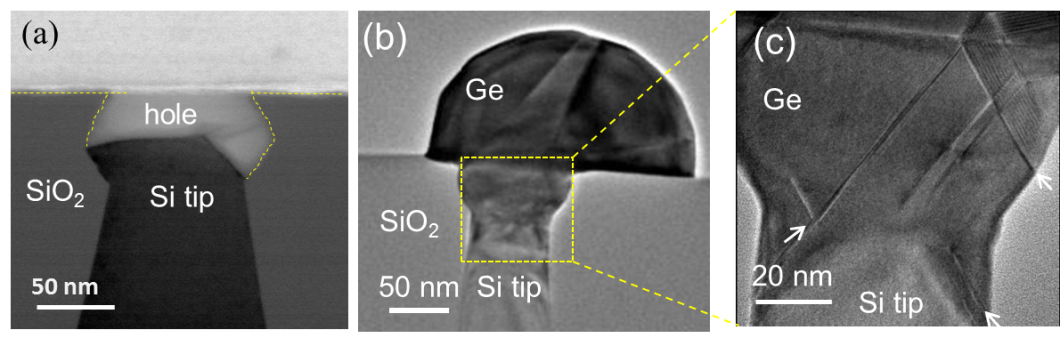


**Figure S6.** Cross-sectional TEM images: (a) a Si-tip substrate after an annealing at 900°C for 5 min in UHV-MBE chamber. The top of the Si tip reacts with surrounding SiO_2_, resulting in a hole and severely deformed Si tip with a irregular shape; (b) Ge island grown on Si-tip substrate shown in (a), which demonstrates many SFs and/or μ-twins; (c) enlarged image of squared region in (b) and the SFs and micro-twins are shown in more details.

**5. TEM statistical analysis**

A TEM statistical analysis carried out on more than 50 Ge islands on the Si-tip wafer, as shown in Figure S7.


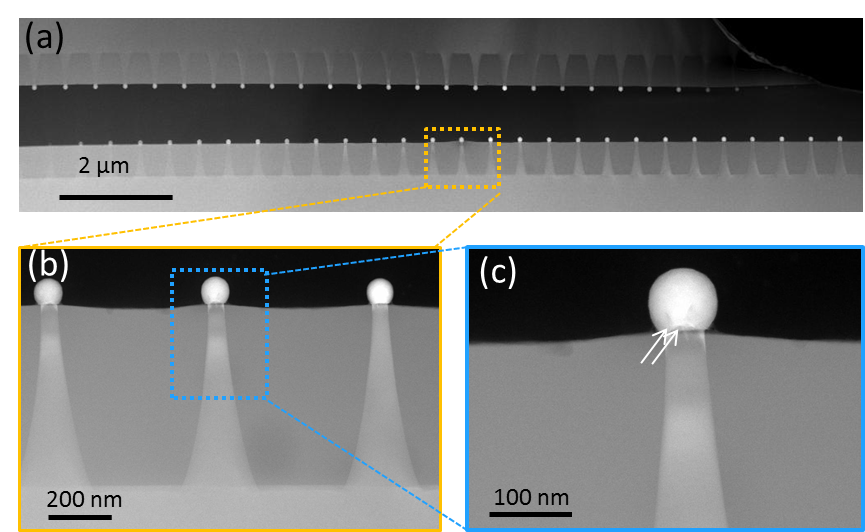


**Figure S7.** STEM images in high angle annular dark field (HAADF) mode for (a) more than 50 Ge islands on the Si-tip wafer; (b) enlarged image of the squared region in (a) with three islands, in which two are defect-free and SFs were observed in the island in the middle; (c) higher resolution image for the island in the middle in (b) and arrows mark the SFs induced by the defects on the SiO_2_ surface around the top of the Si tip.

The majority of the islands show no defect like dislocations, SFs or μ-twins, as shown in the Figure S7 (b) for the two islands located at the side parts. Only in a very few islands, as shown by the Ge island in the middle of Figure S7 (b) and more details in Figure S7(c), some SFs (marked by arrows) propagating from the shoulder of the Si tip were observed, which were formed owing to defects on the SiO_2_ surface.
